# High Throughput Ratio Imaging to Profile Caspase Activity: Potential Application in Multiparameter High Content Apoptosis Analysis and Drug Screening

**DOI:** 10.1371/journal.pone.0020114

**Published:** 2011-05-27

**Authors:** Jeena Joseph, Mahendra Seervi, Praveen K. Sobhan, Santhoshkumar Thankayyan Retnabai

**Affiliations:** Integrated Cancer Research Program, Rajiv Gandhi Centre for Biotechnology, Thycaud PO, Thiruvananthapuram, Kerala, India; Bauer Research Foundation, United States of America

## Abstract

Recent advancement in the area of green fluorescent protein techniques coupled with microscopic imaging has significantly contributed in defining and dissecting subcellular changes of apoptosis with high spatio-temporal resolution. Although single cell based studies using EGFP and associated techniques have provided valuable information of initiation and hierarchical changes of apoptosis, they are yet to be exploited for multiparameter cell based real time analysis for possible drug screening or pathway defining in a high throughput manner. Here we have developed multiple cancer cell lines expressing FRET sensors for active caspases and adapted them for high throughput live cell ratio imaging, enabling high content image based multiparameter analysis. Sensitivity of the system to detect live cell caspase activation was substantiated by confocal acceptor bleaching as well as wide field FRET imaging. Multiple caspase-specific activities of DEVDase, IETDase and LEHDase were analysed simultaneously with other decisive events of cell death. Through simultaneous analysis of caspase activation by FRET ratio change coupled with detection of mitochondrial membrane potential loss or superoxide generation, we identified several antitumor agents that induced caspase activation with or without membrane potential loss or superoxide generation. Also, cells that escaped the initial drug-induced caspase activation could be easily followed up for defining long term fate. Employing such a revisit imaging strategy of the same area, we have tracked the caspase surviving fractions with multiple drugs and its subsequent response to retreatment, revealing drug-dependent diverging fate of surviving cells. This thereby indicates towards a complex control of drug induced tumor resistance. The technique described here has wider application in both screening of compound libraries as well as in defining apoptotic pathways by linking multiple signaling to identify non-classical apoptosis inducing agents, the greatest advantage being that the high content information obtained are from individual cells rather than being population based.

## Introduction

Since the identification of the first caspase in the early 1990s and later when better biological systems for apoptosis study were described, the field of cell death research has taken a giant surge. Today, this has led to the identification of molecular mechanisms of newer forms of physiological cell death other than apoptosis like paraptosis, autophagy, necrosis and necroptosis that cross-regulate each other and have common molecular intermediates in deciding and executing the ultimate cell fate [1]–[6]. Mitochondrial outer membrane permeabilization (MOMP), intermembrane space (IMS) protein release, activation of pro-apoptotic Bcl-2 and caspase family proteins have been identified as key events in the initiation, progression and execution of apoptosis [7]–[12]. But, there is still much that we do not understand about how cell death signaling is fine-regulated particularly, how the signaling within a single cell is coordinated for its ultimate orchestration at the population level. For example, there is no clarity about why cell-to-cell variability in death execution exists and whether the hierarchical and spatiotemporal events are integrated at the cellular level or at the population level [13]–[15]. This is because the techniques of study used thus far have been limited to either single cell studies or population level studies only.

Recent single cell studies using time-lapse confocal microscopy of green fluorescent protein (GFP) tagged apoptotic intermediates like cytochrome C and FRET probes for caspase activation have given valuable insights into the initiation and progression of the hierarchical events of apoptosis signaling within single cells [16], [17], thereby throwing light into the subcellular changes and spatio-temporal relationship between two events. Further, single cell studies have contributed in understanding the ‘point-of-no-return’ or the nature of two different cells that respond differently [18], [19]. Given the advantages, single cell studies are still not sufficient in giving an integrated picture of the subcellular events and the effect of these in a single cell on the population as a whole. This is because, in spite of the fact that single cell studies give real time low-noise information in single cells, concomitant analysis of the response of the whole population is practically impossible using the presently available techniques. On the other hand, population based studies inspite of giving robust results, bear noise as well as fail to give details of subcellular spatial and temporal apoptotic events as in single cell studies.

Basic cancer research has given us remarkable insights into our understanding of the biology of cancer with the realization that apoptosis and the genes that control it have a profound effect on the malignant phenotype [20], [21]. Cancer-generating mutations disrupt apoptosis, leading to tumor initiation, progression or metastasis. The mutations that suppress apoptosis also reduce the sensitivity to cancer treatment [22]. Considering the enormous evidence linking cancer and apoptosis, it appears that screens of apoptosis rather than the cell protein content status are far more superior for anticancer drug screening [23], [24]. However, miniaturization of apoptosis detection screens for highthroughput assays remains a challenge. Caspase activation is an accepted read-out of apoptosis and proteinase activity and can be exploited for biomolecular screening [25]. Caspase activation can be detected by measurement of catalytic activity, immunoblotting, immunolabeling using conformation-sensitive antibodies or affinity labeling followed by flow cytometry. Out of these, live cell real time detection of activity is possible only by a FRET based probe-cleavage study [26], [27]. All the other methods either require cell-free protein extracts to be prepared or cell fixation done, thus being non-adaptable for a live cell high throughput (HTS) study. Current caspase activation HTS assays are based on fluorigenic substrates or FRET based probes, the former being an end-point detection. Also, the FRET based live cell HTS detection systems developed so far have failed to give quantitative information in a cell-to-cell specific, real-time manner, or are not adaptable for multiparameter analysis [28].

We have developed a novel highthroughput platform in cell lines of varying origins that could be adopted for real time, multiparameter apoptosis detection and high content drug screening. To enable simultaneous detection and analysis of multiple parameters using spectrally similar fluorophores, spatially separated segmentation masks were employed. By employing this approach, we have identified novel non-conventional apoptosis induction mechanisms of certain drugs. A high content screening platform to assay multiple death signals like caspase activation, MMP loss, chromatin condensation and mitochondrial superoxide generation simultaneously in live cells as is shown in our study helps to link the interrelationship of these critical events in the execution of cell death in HTS fashion. Through a time kinetics study we found two response groups of ‘sudden’ acting and ‘gradual’ acting drugs. This assay is useful for comparing caspase activation profiles among different cell lines as well as for that of initiator and effector caspase activity in the same cell line itself. Another important advantage of the platform described is long term fate analysis of non-responsive cells. Using this, we report a non-caspase 9 mediated pathway of death induction of 3 classes of compounds in the cell population that survived initial death induction by these same compounds. This is the first ever report of a highthroughput platform which records subcellular signals from individual cells so as to give a more accurate picture of the whole population and thus becomes useful in the real-time tracking of multiple apoptosis parameters in live cells, that can be adapted for direct screening of drugs for multiple cellular signaling.

## Materials and Methods

### Cell culture and drug addition

All cells were maintained in Dulbecco's Modified Eagle's Medium (DMEM, Gibco) containing 10% Fetal Bovine Serum (FBS) and antibiotics in a humidified CO_2_ (5%) chamber at 37°C. For experiments, cells were seeded in 96 well plates at desired densities. After an overnight culture, medium was removed and 100 µl fresh phenol-red free DMEM containing 5% FBS with the compounds to be tested were added. Concentrated stocks of each drug was prepared in either DMSO or water according to solubility and stored at −80°C. The list of drugs used, known mechanism of action and the respective concentration have been given in supplemental information (Table S1).

### Expression vectors and generation of stable cell lines

SCAT3 and SCAT9 vectors were kindly gifted by Dr. Masayuki Miura, University of Tokyo, Japan [30]. The former is linked with preferred caspase 3/7-specific DEVD recognition site and the latter with caspase 9-specific recognition site LEHD in between donor and acceptor. SCAT 8 was supplied by Dr. Markus Rehm, Royal College of Surgeons in Ireland, Ireland that is interconnected with short linker of preferred caspase-8/-10 recognition site IETD between donor and acceptor [16]. Cell transfection was performed using Lipofectamine™ 2000 (Invitrogen, Carlsbad, CA) as per the manufacturer's instructions. Stably expressing clones were generated by selecting the cells in 800 µg/ml of G418 (Invitrogen, Carlsbad, CA) containing medium for 30–45 days. Following clonal propagation, the multiple clones with different levels of transgene expression were expanded and further sorted based on quantitative FRET, analysed by FACS Aria (Becton Dickinson) so as to obtain an enriched population of SCAT3, SCAT8 and SCAT9 expressing cells with optimum default FRET. Briefly, cells were excited with 407 nm laser and the FRET-high cells were identified from the scatter plot of ECFP emission collected using 450±40 nm filter against EYFP emission collected using 530±30 nm filter.

### FRET acceptor bleaching by Confocal laser scanning microscopy

Cells seeded in chambered coverglass (Lab-Tek™, Nunc, Rochester, NY) were sequentially scanned using a laser scanning Leica TCS SP2 Confocal microscope (Leica Microsystems, Germany) for donor (ECFP) and acceptor (Venus) fluorescence signals under the 458 nm and 514 nm laser lines respectively to obtain pre-bleach donor intensity (Donor_pre_) and pre-bleach images. Following this, the acceptor was bleached off completely with high intensity 514 nm laser pulses after which, the donor intensity was obtained (Donor_post_) and FRET efficiency was calculated using the formula: % FRET efficiency = (Donor_post_−Donor_pre_)/Donor_post_. Pre-bleach and post-bleach donor/acceptor intensity images were used to correlate the results visually.

### Flourescence time lapse imaging

Cells were grown on chambered cover glass (Lab-Tek™, Nunc, Rochester, NY), to which, the indicated drugs reconstituted in DMEM containing 5% FBS was added. For imaging, cells were stained as described and imaged in a live cell incubation chamber (Tokai Hit, Japan) that maintains optimum CO_2_, temperature and humidity. Imaging was carried out under a 20× Plan Apo 1 NA objective under an inverted fluorescence microscope (Nikon Eclipse, TE2000-E) equipped with a CARV II confocal imager (Becton Dickinson) either in confocal or non confocal mode and an automated excitation and emission filter wheel. The images were captured using EMCCD camera Andor iXON 885 using IPLab software (BD, USA) at regular intervals for the indicated time periods. To minimize photobleaching, the intensity of the light was reduced to 1% by intensity iris control. For ratio imaging of CFP/YFP, CFP was excited with 436±10 nm band pass filter and CFP emission was collected using 483±22 nm band pass filter. FRET channel was collected using 542±27 nm band pass filter.

### Detection of FRET by flow cytometry

CFP/YFP ratio analysis by FACS was performed in a FACSAria II (BD Biosciences) equipped with a 407 nm laser. Briefly, cells seeded in 12-well plates were treated with drugs. At the end of drug treatment, cell were trypsinized, washed twice with PBS and passed through a 40 µm sieve (BD Biosciences) before FACS analysis. To measure ECFP, cells were excited with the 407 nm laser and fluorescence was collected in the ECFP channel with the standard 450±40 nm filter, while the EYFP FRET signal (for measurement of Venus) was measured with a 530±30 nm filter. For each sample, we evaluated a minimum of 20,000 cells that fell within the background adjusted gate. In the scatter plot of ECFP Vs EYFP, the events that were detected within the gate drawn corresponding to increased ECFP and reduced EYFP were taken as cells with loss of FRET.

### Live cell staining

#### Hoechst 33342 staining

Following drug treatment, treated and untreated cells were stained with 2 µg/ml Hoechst 33342 (Molecular Probes #H1399) in phenol-red free DMEM containing 5% FBS by incubating at 37°C for 10 minutes. Unbound dye was washed off by gently washing twice with PBS. Hoechst 33342 intensity was imaged for detecting pycnotic nuclei. Cells were counterstained with TMRM (Molecular Probes #T-668) or MitoSOX™ Red (Molecular Probes #M36008) after Hoechst staining. **TMRM staining:** For TMRM staining, cells were incubated with 150 nM TMRM at 37°C for 10 mins following which, unbound dye was washed off. Stained cells were replenished with 5 nM TMRM containing 5% phenol red-free DMEM for imaging MMP loss. **MitoSOX Red staining:** MitoSOX™ Red mitochondrial superoxide indicator is a fluorigenic dye for highly selective detection of superoxide in the mitochondria of live cells. For staining, cells were loaded with 5% DMEM containing 5 µM MitoSOX™ Red and incubated for 10 minutes at 37°C, protected from light. Cells were washed thrice gently with PBS to remove excess unbound dye and replaced with fresh 5% phenol-red free DMEM.

### Highthroughput screening: acquisition, segmentation and analysis

#### Image acquisition

For HTS imaging, cells were seeded in 96 well glass bottom plates (Greiner Bio-One) at desired density. After an overnight culture, medium was removed and replenished with phenol-red free DMEM containing 5% FBS with the compounds to be tested at the appropriate working concentrations. Plates were imaged under BD Pathway™ 435 Bioimager (Becton Dickinson Biosciences, USA). The filter combination for CFP/YFP FRET was designed as described for microscopic imaging with independent automated control of excitation, emission and dichroic filter wheels for easy adaptation of CFP/YFP ratio image acquisition with other parameters of choice. The excitation/dichroic mirror/emission filter combinations used for imaging were 438±24/458LP/483±32, 438±24/458LP/542±27, 562±40/593LP/624±40 and 377±50/409LP/435LP for CFP, CFP-YFP FRET, TMRM/MitoSOX and Hoechst channels respectively for multiparmater high throughput image acquisition. Images for each well were acquired in the respective channels using a dry 20× objective with NA 0.75. Images were captured as single montage/3×3/6×6 montages. For caspase inhibition, cells were pretreated for 1.5 hours with the respective caspase inhibitors Caspase inhibitor VI, Caspase 8 inhibitor I and Caspase 9 inhibitor II (Calbiochem) for inihibiting caspase 3, 8 and 9 activities respectively, following which, cells were subjected to drug treatment in the following manner: MCF7 SCAT3 and SCAT9 cells with Staurosporine (250 nM) and SCAT8 cells with Anisomycin (2 µg/ml) for 10 hours. At the end of the incubation time period, cells were subject to imaging for FRET loss positive cells.

#### Post acquisition segmentation and analysis

Post-acquisition image analysis was done using BD AttoVision™ (Version 1.6/435, Becton Dickinson Biosciences, USA) by applying simple polygon segmentation or polygon band segmentation for demarcating cytoplasmic and nuclear regions separately at the automatic threshold level and variable dilation as well as erosion width. Optimized segmentation masks were created for each experiment by an in-built distance-map based split algorithm for separating touching cells with desired erosion factor based on the cell type. Data was read out in the form of images and dot plots, with FRET ratio loss (increased DEVDase activity) corresponding to increased ECFP/EYFP ratio signal, TMRM intensity loss for mitochondrial membrane potential (MMP) loss, increase in Hoechst 33342 intensity for chromatin condensation and MitoSOX intensity gain for mitochondrial superoxide generation.

#### Data export and quantitation

Following automated segmentation and ROI identification, the data was exported to BD FacsDiva™ and gates were applied for the corresponding quantitative read-outs. Gates were made in each experiment keeping basal apoptosis parameter in untreated controls below 10%. Read out of cells with greater FRET loss and chromatin condensation than the set threshold was taken as events in the upper quadrant, whereas, for MMP loss, those in the lower quadrant were considered. For the generation of intensity maps and Z^'^ factor values, data was exported to BD Image Data Explorer™ (Version 2.2.15, BD Biosciences, USA). In order to estimate the ‘screenability’ of the HCS assay, Z^'^ factor values were calculated using the equation Z^'^ = 1−(3*StdDev_sample_+3*StdDev_control_)/(Mean_sample_−Mean_control_).

### SDS-PAGE and Western blotting

50 µg protein sample was resolved electrophoretically in 12% SDS–polyacrylamide gel and electro transferred onto a Hybond C™ extra nitrocellulose membrane (Amersham Biosciences) by wet transfer procedure (Bio-Rad Mini Protean11). The membrane, after blocking with 5% BSA in TBST was incubated with primary antibody overnight at 4°C. The primary antibodies used were: rabbit anti-GFP (#2555, Cell Signaling Technology), mouse anti-caspase 8 (#9746, Cell Signaling Technology), mouse anti-caspase 3 (#9662, Cell Signaling Technology), caspase 9 (#551247, BD Pharmingen), Cleaved caspase 3 (#9661, Cell Signaling Technology) and mouse anti-β actin (#A5316, Sigma) at 1∶1000 dilutions. The membranes were then incubated in horse-radish peroxidase labeled anti-rabbit or anti-mouse secondary antibodies (Sigma) at 1∶4000 dilutions for 1 hr and developed using Enhanced Chemi Luminescence assay (Amersham™ ECL Plus western blotting detection kit, GE healthcare, Buckinghamshire, UK) or DAB (Sigma).

## Results

### Validation of the caspase sensor transfected stable clones for presence and functionality of caspase sensor probe

**Figure 1:**
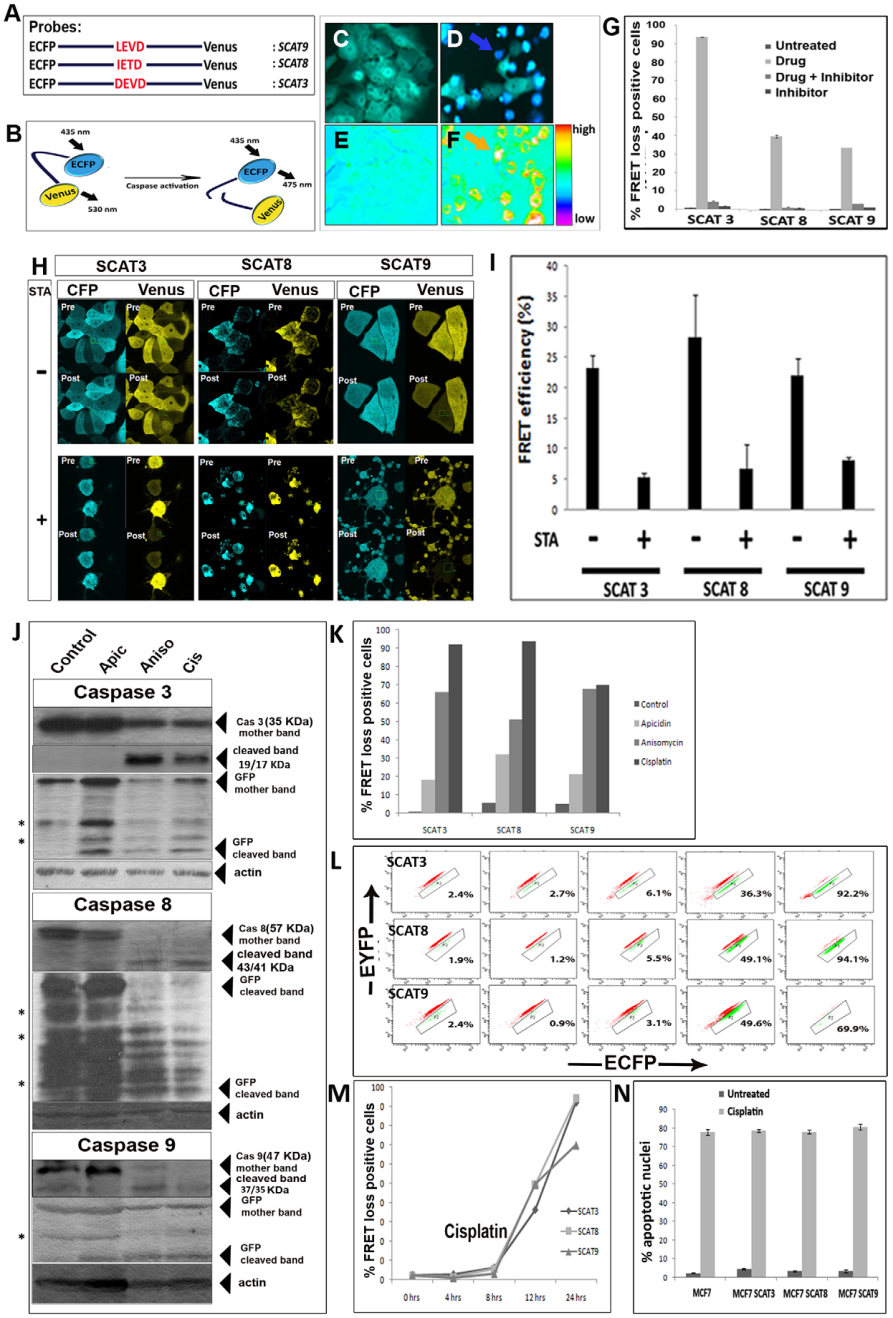
Validation of caspase activity sensor probes. (**A**) Schematic representation of the FRET probes with corresponding caspase substrate-specific linkers. (**B**) The schematic representation of the default FRET between ECFP and Venus in untreated cells and its loss upon linker cleavage is shown. (**C**) Merged image of ECFP and FRET channels of untreated MCF7 SCAT3 cells (**D**) Merged image of ECFP and FRET channels of MCF7 SCAT 3 cells after treatment with staurosporine (500 nM, 12 hrs). (**E**) Ratio pseudo-image of the same cells untreated and (**F**) staurosporine treated cells. Arrows depict a representative cell showing FRET loss. (**G**) Histogram showing reversal of percentage of high FRET loss positive cells upon treatment with the corresponding caspase inhibitor in MCF7 SCAT3, SCAT8 and SCATcells. The cells were subjected to drug treatment in the following manner: MCF7 SCAT3 and SCAT9 cells with Staurosporine (250 nM) and SCAT8 cells with Anisomycin (2 µg/ml) for 10 hours.selected based on the maximal activity observed. Images shown in Supplementary figure S3 (**H**) Confocal-based FRET acceptor bleaching in MCF7 cells showing pre-bleach and post-bleach images of untreated and treated (STA 500 nM, 12 hrs) (**I**) FRET efficiency calculated from confocal acceptor bleaching experiments of cells expressing indicated probes with and without STA treatment. (**J**) Immunoblot of the drug treated HeLa SCAT3, SCAT8 and SCAT9 cells and the corresponding cleavage of endogenous caspases as well as SCAT, probed with anti-GFP (**K**) Percentage of cells with ECFP-EYFP FRET loss analysed for the indicated probe-expressing HeLa cells analysed by FACS after treatment for indicated time points. (**L**) Flow cytometry scatter plot of % cells with high ECFP- EYFP FRET ratio loss of HeLa SCAT3, SCAT8 and SCAT9 cells after 0 h, 4 h, 8 h 12 h and 24 h of Cisplatin (50 µg/ml) treatment: and graphical (**M**) representation. (**N**) Untransfected MCF7 cells and its SCAT3/SCAT8/SCAT9 transfected counterparts tested for % apoptotic nuclei after Cisplatin (50 µg/ml, 10 hrs) treatment.

The image-based high content screening (HCS) methodology described here utilizes intracellular FRET-based caspase activity sensor probes having ECFP and Venus tags as the donor and acceptor fluorophores. A panel of cell lines with the transfected probes was stably generated as described in *Materials and Methods* (Table S2), which were then validated for the presence of the transfected gene product using different methods. Employing fluorescence time lapse imaging, FRET probe cleavage, which corresponded to an increase in cyan coloured (CFP channel) over green colored (YFP channel) cells was taken as the read out for caspase activation (Figure S1, Movie S1) with untreated cells not showing any such change. Once caspase activation was thus qualitatively confirmed by fluorescence imaging (**Figure 1 C–F**), quantitation of the same in single cells was performed by confocal microscope-aided FRET acceptor bleaching method as described in *Materials and Methods*. From this, in MCF7 SCAT3, MCF7 SCAT8 and MCF7 SCAT9 cells, untreated population gave FRET efficiencies of 23.28±2.08%, 28.37±6.94% and 22.10±2.78% respectively, whereas the population treated for 12 hrs with Staurosporine (STA) gave reduced FRET efficiencies of 5.32±0.69%, 6.7±4.04% and 8.21±0.40% in the respective cell variants. Caspase inhibitors for the three caspases reversed the FRET loss information as seen by the reduction in FRET loss positive cells (**Figure 1 G**) (Supplementary Fig. S3). In order to confirm whether SCAT probe cleavage was comparable to the corresponding endogenous caspase activation pattern, we probed samples of HeLa cells treated with Apicidin, Anisomycin and Cisplatin for caspase full length protein cleavage as well as SCAT probe cleavage. The results displayed similar activation profiles between the two, thereby confirming the utility of the probes as a direct read-out for the endogenous caspase activation; SCAT3 for DEVDase, SCAT8 for IETDase and SCAT9 for LEHDase activity (**Figure 1 J**). Further, using flow cytometer-based FRET detection, we confirmed the results obtained through western blotting (**Figure 1 K**). Also, a kinetics analysis of the percentage of cells showing FRET loss upon treatment with few drugs (data of Cisplatin shown) for SCAT3/8/9 probe cleavage was done by flow cytometry, which showed a gradational activation pattern over a time period of 0 to 24 hrs studied, with a surge of activation after 8 hrs post treatment (**Figure 1 L,M**). This shows that the panel of cell lines developed could also be used to study kinetics of percentage of cells with active caspases over a period of time. Also, in order to address the issue of transfectioninduced artifacts in the apoptotic response, we looked for the percentage of apoptotic nuclei in untransfected and transfected cells (**Figure 1N**). It is noteworthy that cell reponse does not significantly vary in both the cases. All these methods thus confirmed the functionality of the probes in the stable cell lines generated both qualitatively as well as quantitatively.

### The HCS platform is adaptable for high content apoptosis analysis

**Figure 2:**
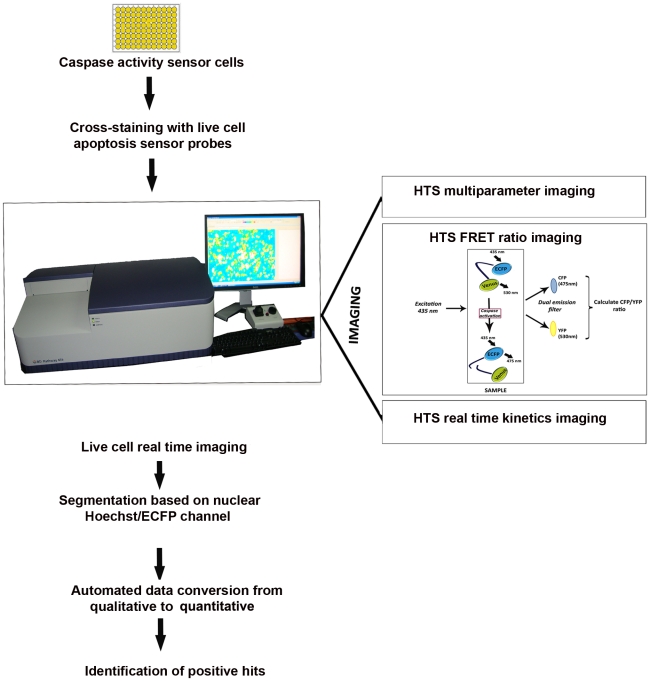
Schematic overview of the high-content imaging methodology. The method utilizes 96 well plate ratio imaging as the primary readout of caspase activity in cells stably expressing caspase cleavable FRET probe as described earlier. The pathway Bio imager was configured with ECFP and EYFP FRET filter as described in the materials and methods. The cells were seeded on 96 well glass bottom plates at the desired density and image acquisition was done in an automated on-the-fly ratio mode with desired montage option at regular time intervals. Post acquisition analysis was done using Attovision software after automated segmentation into nuclear and cytoplasmic channels, followed by conversion of qualitative data to quantitative data. The quantitative ROI information is converted in to list mode that can be analysed in any flow cytometer based software. The cumulative information helps in the identification of positive hits.

**Figure 3:**
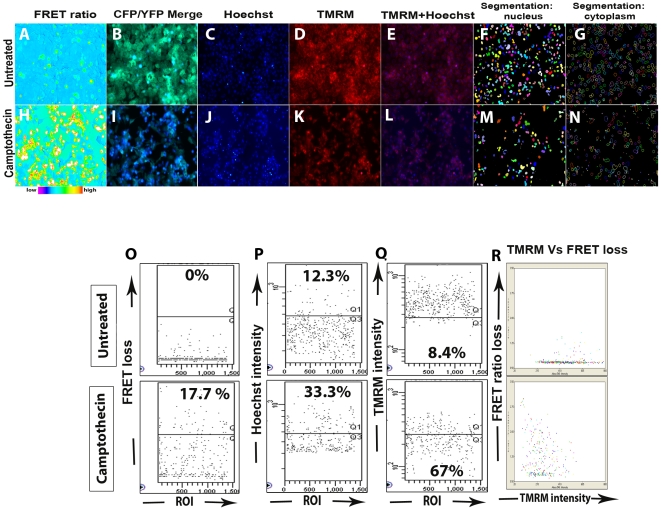
A representative output data with multiparmater analysis. A representative output data from OVCAR8 SCAT3 cells showing FRET loss with increase in donor blue fluorescence upon drug treatment in the CFP/YFP merge channels (**B,I**) with the corresponding FRET ratio pseudo-color channel (**A, H**) TMRM (**D,K**) and Hoechst fluorescence channels (**C,J**) (untreated: **A–E**, Camptothecin (10 µM, 12 hrs) treated **H–L**). Segmentaion mask (polygon band) showing spatial demarcation between nucleus and cytoplasm for both untreated (**F&G**) and camptothecin (10 µM, 12 hrs) treated (**M&N**) are also shown. The corresponding post-analysis quantitation carried out by FACS Diva software after indicated treatment is shown (**O–R**). Percentage of desired apoptosis parameter are shown in the corresponding gated areas FRET loss (**O**), hoechst intensity (**P**) and TMRM intensity (**Q**). Dot plot showing biparameter analysis of TMRM intensity Vs FRET loss (**R)**.

**Figure 4:**
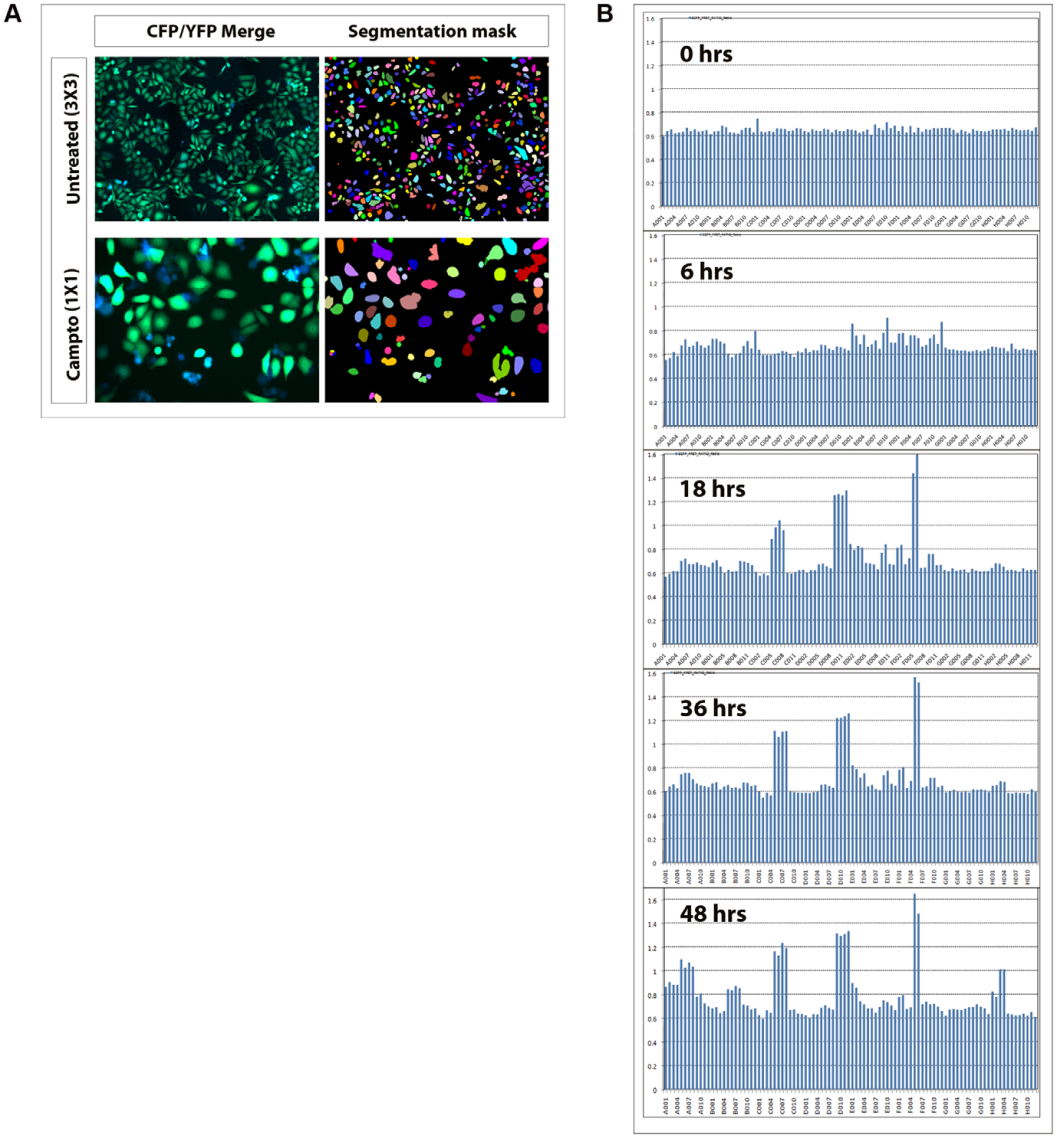
The HCS platform is adaptable for high content apoptosis analysis. OVCAR8 cells treated with a random panel of drugs in duplicates were imaged at the time points: 0 hr, 6 hr, 18 hr, 36 hr, 48 hr post drug treatment. Simple polygon segmentation was applied (**A**) and segmentation parameters were optimized to pick out intact cells as well as apoptotic bodies. Montages of 1×1 and 3×3 are shown to highlight the possibility of choosing different montage types based on the type of experiment or to increase the number of cells for analysis. Bar chart showing time kinetics of FRET ratio loss upon drug treatment at the indicated time points (**B**). The same area and same cells are represented over a period of time indicating potential for readout of kinetics.

Once validation of the engineered cells were complete, a platform for high content imaging and apoptosis analysis using these cell lines was designed (**Figure 2**). A representation of the output data is also shown (**Figure 3**). First, in OVCAR8 SCAT3 cells we used a random panel of drugs (in duplicates) from that given in *Supplementary* information to check for the adaptability of the method for high content screening. For this, cells were imaged at 0, 6, 18, 36 and 48 hrs post drug treatment. Data was analysed using polygon segmentation, where, FRET signal was measured in the whole cell. By the segmentation applied, neighboring cells could be clearly demarcated and apoptotic bodies as well as spread out cells could be picked up together for analysis. The cells imaged with 3×3 montage acquisition as well as single montage image with respective segmentation masks are represented in **Figure 4A**. In general for most comparative data generation, cells were imaged with 3×3 montage to cover large population of cells from each well. In the pattern of drug activity obtained, some drugs were found to be active very early after drug treatment, while others displayed a slow, gradational activation (**Figure 4B**). Slight caspase activation could be picked up even as early as 6 hrs post treatment, which could be neatly followed up upto the 48 hrs time point analysed. Thus, it was confirmed that this could be adapted as a sensitive and reliable assay methodology for high content caspase activation profiling of live cells.

### The HCS platform is adaptable for multiparameter apoptosis analysis

**Figure 5:**
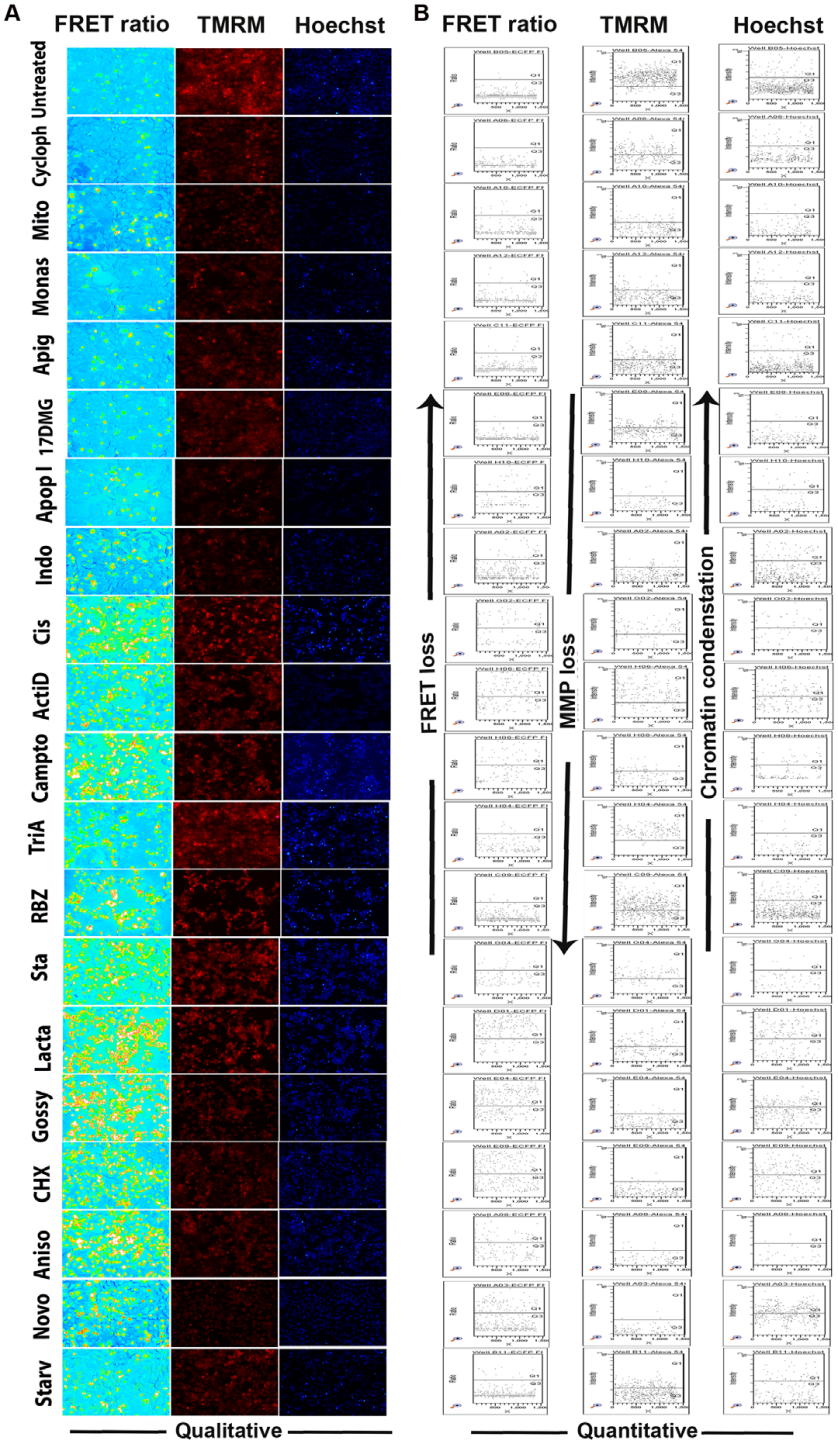
High content multiparameter analysis: FRET ratio loss, TMRM intensity and chromatin condensation. OVCAR8 cells stably transfected with SCAT3 was treated with 42 drugs (in duplicates) for 20 hrs. Following drug treatment, cells were imaged for FRET ratio loss, TMRM loss and chromatin condensation under the BD pathway imager as described in *Materials and Methods*. Polygon band segmentation was applied and the 19 positive hits obtained displaying variable MMP loss, FRET ratio loss and chromatin condensation response patterns are shown. (**A** Images of FRET ratio loss, TMRM and chromatin condensation are shown, (**B**) with the corresponding scatter plot representation obtained through data export and analysis in BD FACSDiva software.

Since a high content screening platform to assay multiple apoptosis parameters like caspase activation, MMP loss and chromatin condensation simultaneously in live cells has not been reported yet, we wanted to check for the adaptability of this platform for such a multiparameter apoptosis analysis. Initially spinning Disk confocal imaging was carried out to check the suitability for multiparamater imaging of MMP loss together with ratio analysis for caspase activation status in live cells. MCF7 SCAT 3 cells treated with Actinomycin D after staining with MMP indicator TMRM were imaged using CARV confocal imager by time lapse as described. The representative images from multiple time points and video of merged channels are shown in **Figure S2, Movie S2**. As seen from the figure and movie, temporal information of loss of TMRM fluorescence and FRET can be easily monitored visually. For high throughput analysis, OVCAR8 cells stably transfected with SCAT3 was treated with 42 drugs (in duplicates) for 20 hrs. Following drug treatment, cells were imaged for the above-mentioned parameters under the BD pathway imager as described in *Materials and Methods*. We obtained, among these, 19 positive hits displaying variable MMP loss, FRET ratio loss and chromatin condensation response patterns depending on the mechanism of action of the drug (**Figure 5**). From the results, even at 20 hrs post treatment, the drugs Cyclophosphamide, Mitoxantrone, Monastrol, Apigenin, 17 DMAG, Apoptosis Activator I and Indomethacin displayed only a loss in mitochondrial membrane potential (MMP loss) that was not accompanied by a correspondingly elevated caspase activation or chromatin condensation. DNA damaging agents Cisplatin, Actinomycin D, and Camptothecin, HDAC inhibitor Trichostatin A, kinase inhibitors 5,6-dichloro 1,β-D ribofuranosyl benzimidazole and Staurosporine and Lactacystine were found to induce FRET ratio loss without a correspondingly massive MMP loss. This points towards a Δψ_m_- independent DEVDase activity possible with drugs that induce damage of DNA or associated machinery. On the other hand, FRET loss, MMP loss and chromatin condensation were all found to be induced by translation inhibitors Cycloheximide, Anisomycin as well as Novobiocin and Gossypol at 20 hrs post treatment. Furthermore, starvation (0.5% serum in culture medium HBSS) condition for 20 hrs induced chromatin condensation and slight reduction in MMP, without significant caspase activation.

**Figure 6:**
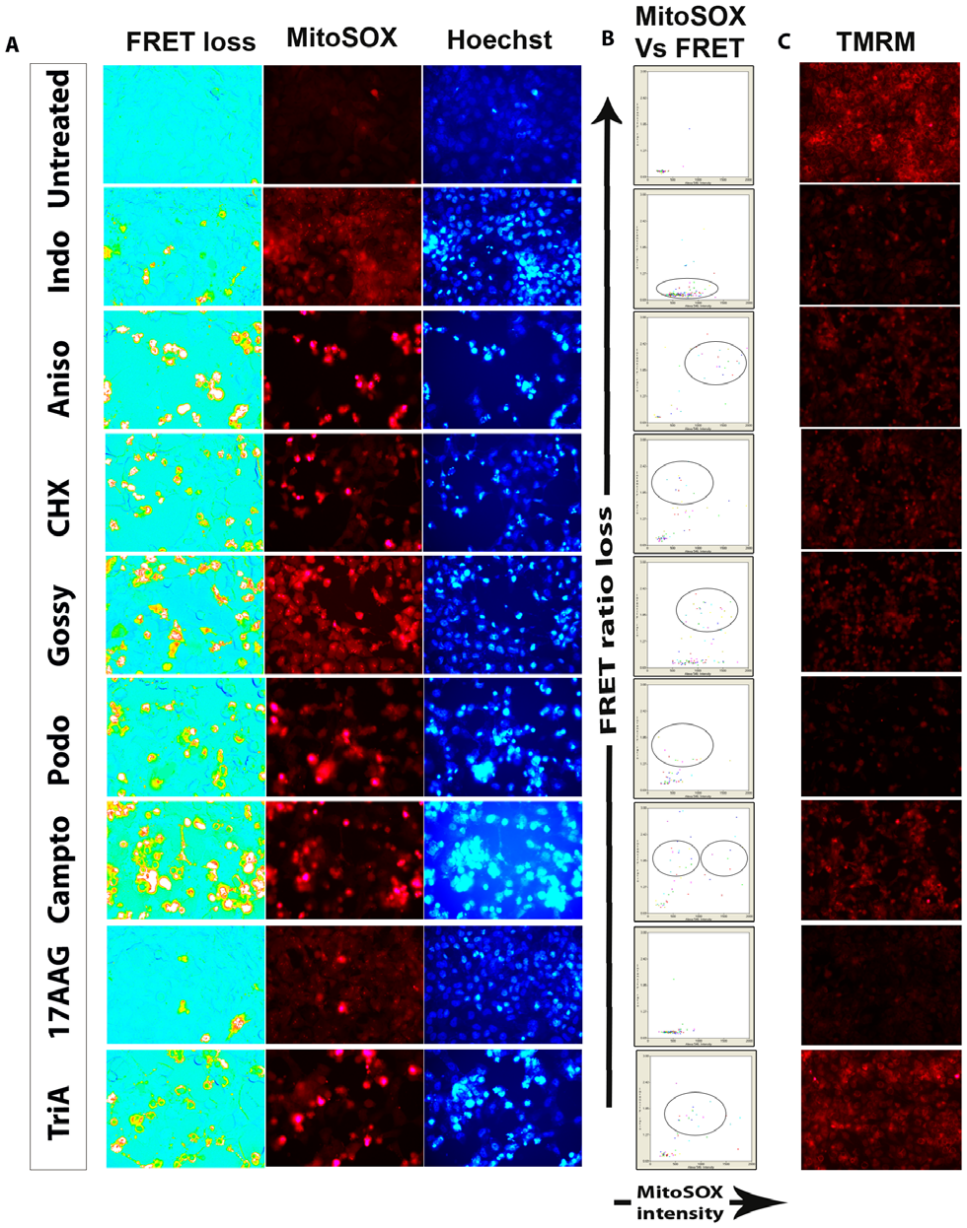
High content multiparameter analysis: FRET ratio loss, TMRM intensity, chromatin condensation and MitoSOX production. For simultaneous imaging and correlation between mitochondrial superoxide (SOX) generation and MMP loss along with FRET ratio loss together with chromatin condensation in the same cells, OVCAR8 SCAT3 cells were stained in separate wells with mitochondrial SOX indicator dye MitoSOX Red and TMRM dye. 8 positive hits were selected on the basis of robust variations (**A**) Images of FRET ratio loss, mitochondrial superoxide and chromatin condensation from the same well and the (**C**) corresponding TMRM pattern from a different well (**B**) Biparametric dot plot showing comparison of MitoSOX and FRET ratio loss. Encircled area shows the response patterns with each drug.

Further, in order to look at the correlation between mitochondrial superoxide (SOX) generation and MMP loss along with FRET loss and chromatin condensation in the same cells, we stained separate wells with mitochondrial SOX indicator dye MitoSOX Red and TMRM dye. Eight positive hits were selected on the basis of robust variations (**Figure 6**). We found a high correlation between MitoSOX gain and TMRM loss in all the drugs tested, except for Gossypol, which induced considerably very high SOX production when compared to the MMP loss. Our multiparameter HTS results indicate that the growth inhibitory and toxic activities of Gossypol without a correspondingly massive MMP loss could be due to the massive superoxide generation and an alternate superoxide-mediated, MMP loss-independent apoptosis signaling mechanism. To highlight the multiparameter potential of the method, chromatin condensation and FRET ratio loss channels of the same experiment have also been shown. Overall, the results highlight the potential of the platform to identify non-classical death signaling induced by chemical entities in a high throughput manner with high content multiparameter analysis.

### Adaptability for assaying multiparameter kinetics

**Figure 7:**
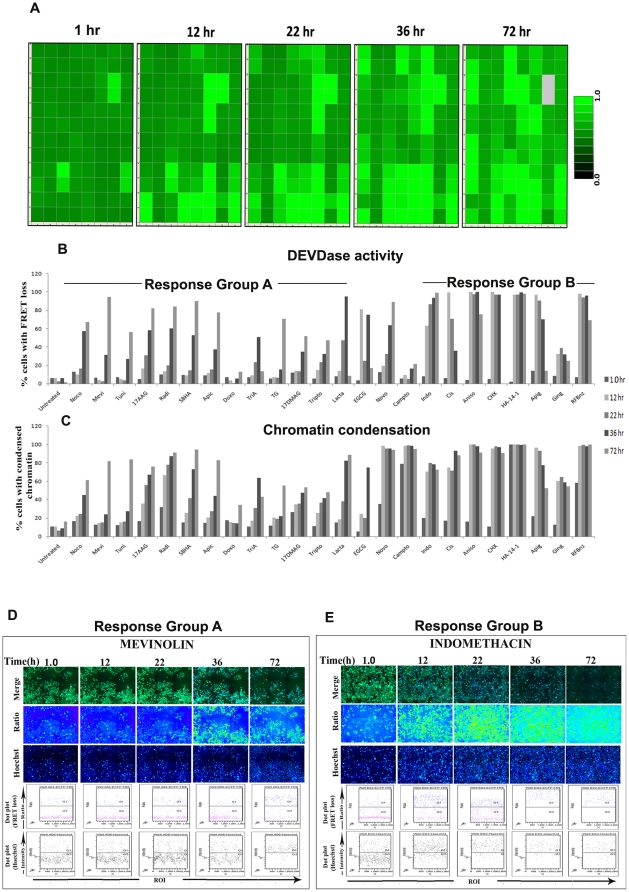
Adaptability for assaying multiparameter kinetics. For assaying DEVDase activity as a function of time, HeLa SCAT3 cells were treated with a panel of 42 drugs (in duplicates) and imaged at the time points: 1 hr, 12 hr, 22 hr, 36 hr, 72 hr post drug treatment for both DEVDase activity and chromatin condensation. (**A**) Heat map of kinetics of FRET ratio loss at the indicated time points. Quantitation of the same was used to plot bar charts for (**B**) DEVDase activity (**C**) and chromatin condensation at the indicated time points, showing 2 response groups with 24 positive hits. Mevinolin and Indomethacin, representative drugs of the two response groups are shown (**D, E**) with upper panel showing CFP/YFP merge, FRET ratio loss and chromatin condensation images and lower panels showing dot plot representations of FRET ratio loss and chromatin condensation at the indicated time points. Details of well positions of drugs used are given in Supplementary Figure S4. Additional details of drug concentration, known mechanism of action have been given in Supplementary Table 2.

For assaying DEVDase activity as a function of time, HeLa SCAT3 cells were treated with a panel of 42 drugs (in duplicates) and imaged at specified time intervals over a period of 0 to 72 hours post drug treatment. Both DEVDase activity and chromatin condensation were assayed in these cells over time. Heat maps depicting the drug response helped identify 24 positive hits that showed gradational DEVDase activity over time (**Figure 7**). Out of these, 12 hits showed very gradual increase in both DEVDase activity and chromatin condensation kinetics, which is depicted as ‘Response group A’, while 7 showed very high activity even as early as 12 hrs after drug treatment, depicted as ‘Response Group B’. A reduction in the read out by 72 hrs in some of the populations is due to the loss of cells in the wells because of excessive cell death. A representative image set and quantitative information of the two response groups have been shown. Our results show that a similar kinetics response pattern is observed among compounds with a common mechanism of action. Hsp90 inhibitors 17AAG, 17DMAG and Radicicol showed a slow, but consistent effect in inducing DEVDase activity and chromatin condensation. Similarly, HDAC inhibitors SBHA, Apicidin and Trichostatin A as well as ER stress inducers Tunicamycin and TG also showed a similar effect. On the other hand, DNA Topoisomerase inhibitors Camptothecin and Novobiocin that directly inhibit the DNA caused massive chromatin condensation at 12 hr itself, but with only a slower effect on the DEVDase activity over time. Translation inhibitors Cycloheximide and Anisomycin, flavonoids Apigenin and Gingerol, Bcl-2 antagonist HA-14-1 as well as CK1 & CK2 inhibitor 5,6-dicloro 1,β-D ribofuranosyl benzimidazole had equal effect on inducing rapid DEVDase activity and chromatin condensation as early as 12 hr after drug treatment. Multiparameter death signaling signatures obtained with drugs of differing mechanisms of action points to the fact that apoptosis centered algorithms can be developed for profiling large chemical libraries for unknown functions.

### Adaptability for comparing differential drug sensitivity among cell lines

**Figure 8:**
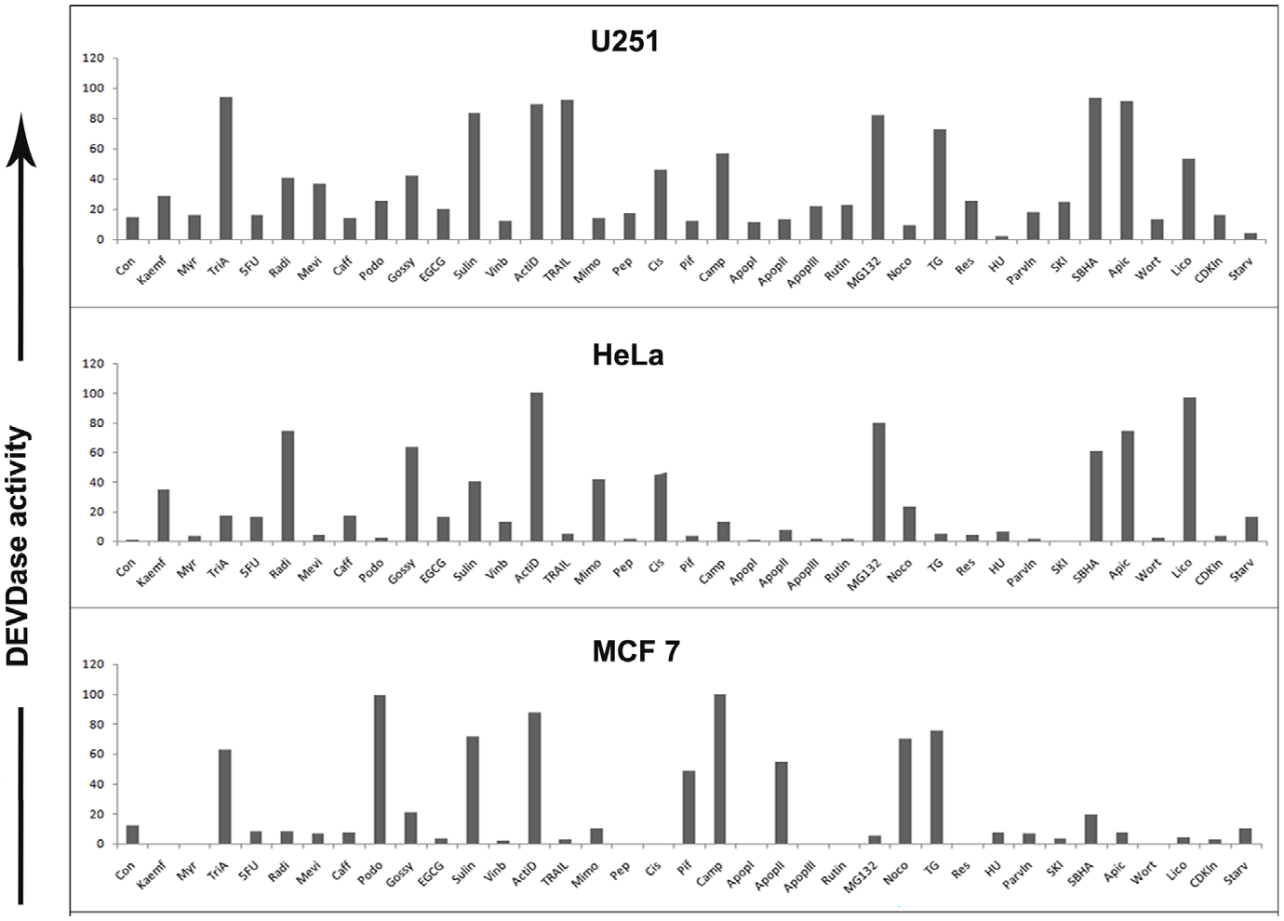
Adaptability for comparing differential drug sensitivity among cell lines. To check for adaptability of the method to compare for differences among cell lines, stably generated U251 SCAT3 and HeLa SCAT3 cells were treated with a panel of 37 drugs (in duplicates) and the DEVDase activity was imaged and analysed at 48 hrs post treatment. 29 hits were identified that showed significant variation from untreated controls. (**A**) U251 SCAT3 and (**B**) HeLa SCAT3 cells are shown.

Response to a particular drug varies from one cell type to another. We wanted to check the adaptability of our method to compare such differences among cell lines. For this, we generated SCAT3 transfected U251 and HeLa stable cell lines, which were treated with a panel of 37 drugs (in duplicates) and the DEVDase activity was imaged and analysed at 48 hrs post treatment. The differential response pattern showed that glioblastoma U251 cells were responsive to a greater range of compounds under the study, when compared to cervical adenocarcinoma HeLa cells (**Figure 8**). U251 cells were most sensitive to Trichostatin A, Sulindac, Actinomycin D, TRAIL, SBHA and Apicidin followed by Radicicol, Mevinolin, Gossypol, Camptothecin, MG132, TG and Licochalcone. HeLa cells showed most sensitivity towards Actinomycin D and Licochalcone, moderate sensitivity towards Radicicol, Gossypol, SBHA and Apicidin, followed by Sulindac and Mimosine. Taken together, Actinomycin D was found to be inducing DEVDase activity both the cell lines under study, although with differing strengths.

Our result reinforces the idea of differential sensitivity of different cell lines to a set of drugs and thereby projects itself as a useful tool in the initial screening for identifying the best set of apoptosis inducing compounds before delving into the mechanism of action in a particular cell line.

### Comparison of IETDase, LEHDase and DEVDase activity against MMP loss

**Figure 9:**
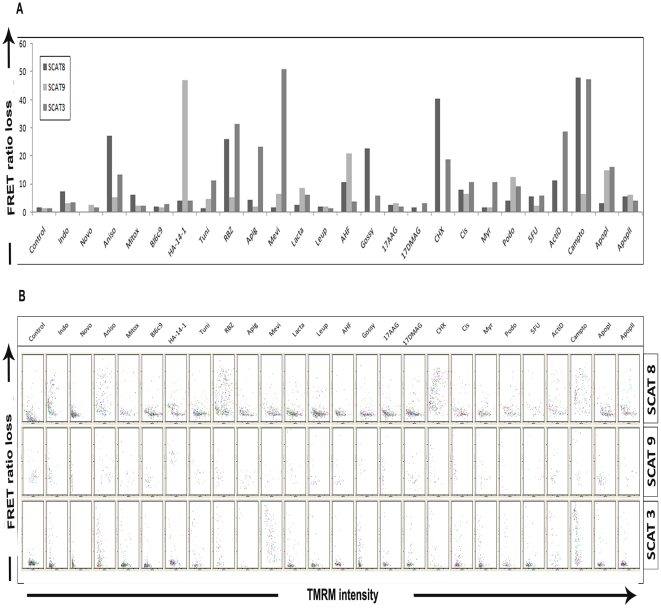
Comparison of IETDase, LEVDase and DEVDase activity against MMP loss. Hela cells stably expressing SCAT3, SCAT8 and SCAT9, after 12 hrs treatment with a panel of drugs were imaged for IETDase, LEHDase and IETDase activity (**A**) Qualitative data was quantified and is represented as bar chart. SCAT3, SCAT8 and SCAT9 FRET ratio loss are represented side-by-side for comparison (**B**) Corresponding biparameteric dot plots showing comparison of IETDase, LEHDase and IETDase activity against MMP loss are shown. The drugs shown here are positive hits obtained from a larger panel of drugs used.

Next, we wanted to make a simultaneous comparative study of IETDase and LEHDase activity together with DEVDase activity and its correlation with Δψ_m_. For this, we generated stable clones of HeLa cells transfected with SCAT3, SCAT8 and SCAT9, which gave a direct read out of DEVDase, IETDase and LEHDase activity respectively. At 12 hrs after drug treatment, as expected, we found considerable variability in the induction of probe cleavage by different drugs (**Figure 9**). We found Cycloheximide, Anisomycin, 5,6-dicloro1,β-D ribofuranosyl benzimidazole (RBZ), Gossypol and Camptothecin to be most active in inducing IETDase activity and HA-14-1, 4-aminohydroxyflavone and Apoptosis Activator I in inducing LEHDase activity. Most of the drugs induced DEVDase activity, the most prominent of those being Apigenin, Mevinolin, RBZ, Cycloheximide, Actinomycin D, Camptothecin, Apoptosis Activator I and Anisomycin. When comparing the probe cleavage with MMP loss, we found a good correlation of LEHDase activity with MMP loss when compared to IETDase activity that was largely not correlated with MMP loss. The DEVDase activity was always accompanied with MMP loss, except in the case of Mevinolin where, FRET ratio loss happened without much reduction in the MMP loss. Thus, this screening platform integrates signals of multiple parameters associated with apoptosis signaling from individual cells so as to generate a final picture of the response of the population as a whole.

### Drug resistant cells execute caspase 9 independent cell death

**Figure 10:**
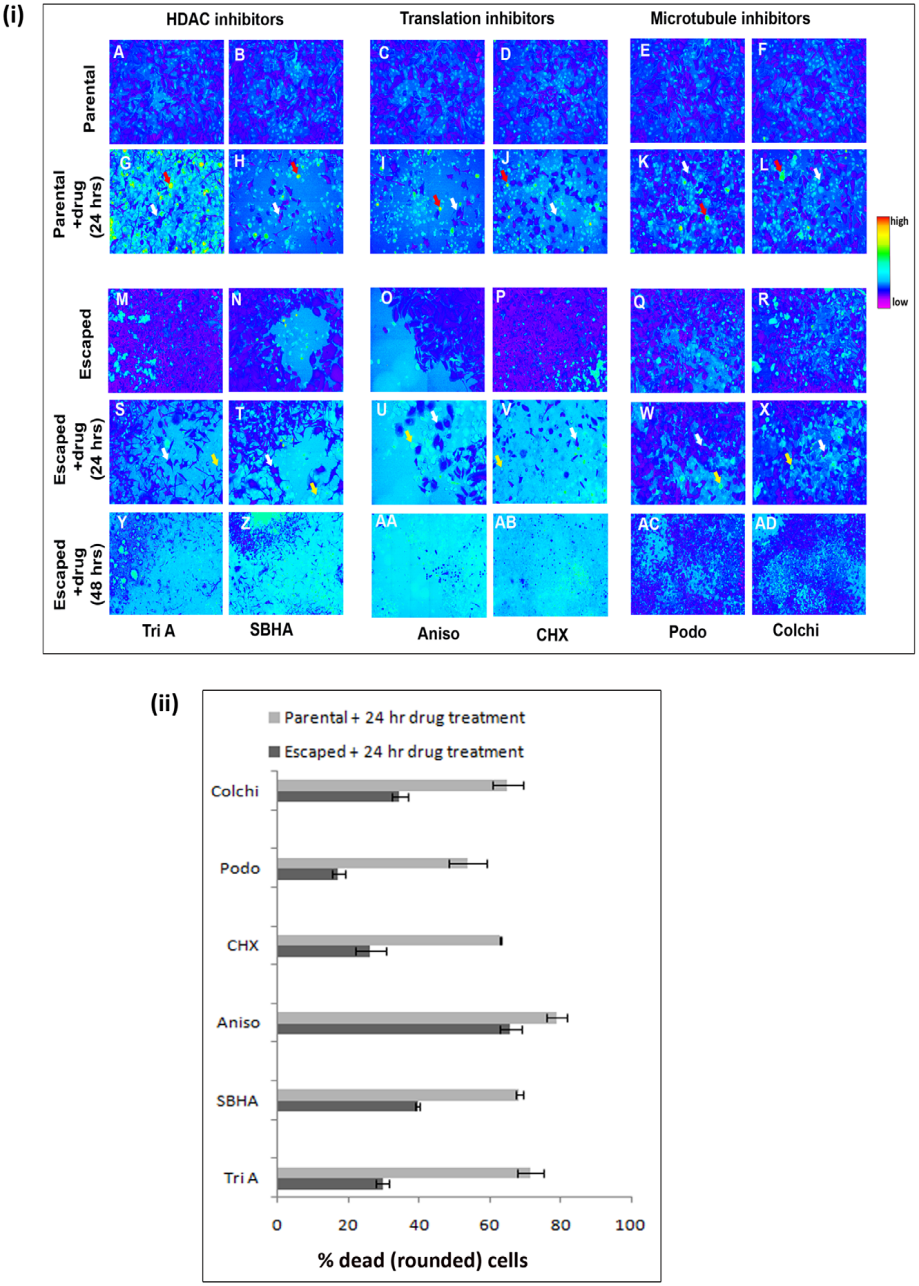
Drug resistant cell execute caspase 9 independent cell death. (**i**) HeLa SCAT9 cells were treated with a large panel of drugs and subject to revisit imaging of the surviving cells. Panels **A** to **F** show parental cells just prior to drug treatment. **G** to **L** represent cell response 24 hrs after the first round of treatment with the indicated drugs. Here, white and red solid arrows indicate representative live adherent (FRET loss negative) and dead rounded (high FRET loss positive) cells respectively. **M** to **R** show colonies out of cells that survived the first round of drug treatment. **S** to **X** and **Y** to **AD** show the response of the same area of the surviving population after 24 hrs and 48 hrs respectively upon second treatment of the escaped population with the same drugs. In panels S to X, white and yellow solid arrows indicate representative live adherent (FRET loss negative) and dead rounded (high FRET loss negative) cells respectively. Heat map shows the representative qualitative low to high FRET loss index (**ii**) Histogram showing percentage of HeLa SCAT9 cells with detached rounded morphology (dead cells) of parental population (light grey solid bars) and escaped population (dark grey solid bars) at 24 hrs post drug treatment with the 6 indicated drugs belonging to 3 classes of mechanism of action.

One of the greatest features of the multi-well plate imaging tool used here is the option of revisit imaging of the same well for longer periods of time, thereby enabling precise fate analysis of the surviving fraction. We identified cell colonies that survived drug treatment in the first round of treatment with 3 classes of drugs viz HDAC inhibitors (TriA, SBHA), translation inhibitors (Aniso, CHX) and microtubule inhibitors (Podo, Colchi) (**Figure 10 i**). When these surviving cells were subjected to a second round of treatment with the same drugs, we found that they took a longer time to respond, and showed a divergent caspase 9 activation response. At 24 hrs post drug treatment, cells that survived after treatment with microtubule inhibitors remained more resistant to a second phase of drug treatment; compared to HDAC inhibitors and translation inhibitors. For comparing death reponse, we scored for the percentage of cells that lost normal growing adherent morphology (rounded cells), treating these as dead cells (**Figure 10 ii**). The population that survived the first round of drug treatment showed a significant reduction in the percentage of dead cells at 24 hrs when compared to the same period of drug treatment of the parental population. The escaped population also exhibited a lesser level of caspase 9 activation in the rounded cells. This is clearly evidenced in the shown FRET ratio heat images by the absence of high FRET loss positive (orange) rounded cells in the escaped drug treated population, when correlated with the representative qualitative heat map.

## Discussion

One of the earliest reports on the potential of GFP-FRET based caspase activation detection for anticancer drug screening in live cells came out in 2000 [26] with several improvisations of this method in the coming years such as advancements in fluorescence signal strength, stability and sensitivity [29]–[31]. Since the readout is from these assays are from well based integrated signal after meticulous background correction, they have limitation in multi-parameter pathway defining or deriving the single cell information. The HTS screening platform described here has several advantages starting from improved stability of probes used to adaptability for high content multiparameter apoptosis analysis in live cell mode. In comparison with the end point, fixed cell, cell proliferation assays used in conventional anticancer drug screening, this platform gives a more physiologically relevant mechanistic picture of the drug activity in the initial screening stage itself. The possibility of on-the-fly acquisition-cum-segmentation-cum-analysis also highlights its superiority over other drug screening platforms. Also, the methodology is simple, in comparison to the enormity of information that is obtained. Further, as tumor etiology varies from one carcinoma type to another, stable generation of these sensor probes in a large panel of cells will enable for comprehensive cell-to-cell comparative study in a kinetic mode. Z factor, which defines the screenability of any particular highthrouput assay, was found to be above 0.7 in all our experiments and for the entire cell lines used, thus demonstrating the usability in large compound library screening.

The development of novel molecularly targeted cancer therapeutics remains slow and expensive with many late-stage failures. This is because most of the frequently used anti-cancer therapies were discovered based on their anti-proliferative activity or cytotoxicity as end point detection in cell-based assays, with no pre-existing knowledge of their targets of action. This kind of a unified approach in drug discovery fails in revealing potential anticancer agents with varied modes of action as a result of which, none of the currently used drugs selectively and directly target the molecular lesions responsible for malignant transformation. Most importantly, considering the enormous evidence linking cancer and apoptosis, it appears that screens of apoptosis rather than anti-proliferative activity are far more superior screens for anticancer drug development. Accordingly, the present study set forth to develop a rational strategy based on live cell apoptosis detection as a useful tool to obtain a better mechanistic understanding of newer anticancer agents. The major highlight of this study is that the conclusions drawn on the response of the population are actually derived by recording the response of each single cell. Therefore the results bear very low noise. Interestingly, soon after drug addition, some wells showed a high DEVDase read out due to the intrinsic fluorescence interference of the drug itself, which remained equally intense upto the assay end point. To the advantage of this methodology, such a display makes the technique suitable for early detection of any spurious signal, thereby preventing the possibility of wrongly interpreting an otherwise negative hit.

Before designing the HTS methodology, we validated the functionality of the probes in the stably generated cell lines by live cell imaging based fluorescence microscopy, FRET acceptor bleaching by confocal microscopy, immunoblotting for FRET probe cleavage and corresponding caspase activation as well as by flow cytometry. We found the cleavage pattern of the transfected probe to be corresponding to that of the activation of the endogenous caspases, thereby showing their suitability for use in further experiments to assay caspase activity. In addition, since there was no significant variation in the percentage of apoptotic nuclei in the untransfected and transfected cell lines, the possibility of transfection-induced artifacts can be ruled out. In fact, we carried out mild selection and flow sorting, thereby being less invasive in enriching the transgene-containing cells, specifically to address this concern. With a simple methodology and using stably generated caspase sensor cell lines, we then developed a high content screening platform that could be adaptable for live cell multiparameter imaging, time kinetics study and cell line-to-cell line comparison of various apoptosis parameters such a mitochondrial membrane potential (MMP) loss, chromatin condensation, mitochondrial superoxide generation together with caspase activation. From our studies, we demonstrate the potential of this high content screening platform in giving integrated information of the varied effects of different drugs based on their mechanism of action. We propose that multiparameter death signaling signatures obtained with drugs of differing mechanisms of actions could be used for developing apoptosis centered algorithms for profiling large chemical libraries for unknown functions.

Upon comparing Δψ_m_ and mitochondrial superoxide (SOX) generation, we found a high correlation between the two in all the drugs tested, except Gossypol, which induced considerably very high SOX production when compared to Δψ_m_. Gossypol, a polyphenolic aldehyde is known to induce Bax/Bak independent cytochrome c release by mediating a conformational change in Bcl2 [32]. In addition, Gossypol has been shown to be mediating free radical toxicity in isolated rat islet cells [33], oxygen radical formation in rat liver microsomes and human sperms [34] as well as ROS mediated growth inhibition in prostate cancer cells. Our multiparameter HTS results indicate that the growth inhibitory and toxic activities of Gossypol without a correspondingly massive MMP loss could be due to the massive superoxide generation and an alternate superoxide-mediated, MMP loss-independent apoptosis signaling mechanism.

Our observation of certain cells that maintain MMP loss without caspase activation or chromatin condensation at a late stage post-drug treatment suggest that the actual ‘point-of-no-return’ of apoptosis is a much more complex event that could probably be ultimately dependent on the mode of action of the death inducer. Also, through this method, compounds that activate caspases independent of the mitochondria-dependent pathway could be detected.

So far, simultaneous detection of apoptosis and mitochondrial superoxide production in live cells worked best with flow cytometry and confocal microscopy [35], which suffer from the disadvantage of either being cumbersome or time-consuming for large scale compound screening. Here we report the first ever comparison of quantitative data on mitochondrial superoxide generation and caspase activity derived from live single cell imaging and thus project the possibility of this methodology in large scale compound screening and generation of multiparameter cell signaling information with a single experiment.

In our multiparameter kinetics analysis, we got two ‘response groups’ of drugs, with one showing gradual activation and the other showing sudden activation. Also, we have compared the IETDase, LEVDase and DEVDase activity against MMP loss in HeLa cells, thereby showing the advantage of this screening platform in giving a comprehensive picture of initator and effector caspase activation in the same cell line. A favourable improvement of this could be the use of a three chromophore FRET based system using for e.g, CFP-YFP-mRFP for the simultaneous detection of multiple caspases or other enzymes within the same cell [36]. Another major advantage of this method is the possibility of following up of the respective wells for subsequent fate, whereby we have identified rare escape of cells from caspase activation with certain antitumor agents. In a unique experiment, we could also show emergence of drug-escaped colonies and their subsequent response to a second cycle of drugs. Employing such a technique, we show that cells that escaped after treatment with cell cycle inhibitors stay more resistant to subsequent re-treatment compared to HDAC inhibitors, translation inhibitors. Most of the stable cell lines described here were clonally expanded and their stable expression have been followed up for more than 200 passages without any significant loss of function and expression. Therefore, they can form a ready-to-use method for HTS-based drug screening applications. However, if this needs to be adopted for other cell lines, stably integrated colonies with optimum FRET efficiency need to be generated as described here. As an extension to our results, we also suggest a future possibility of stable generation of sensor probes in the entire NCI cell line panel with simultaneous multiparameter signaling analysis will help to define the pathway signatures of each drug and may augment selection of best hits from the early screening effort. This is also expected to augment the identification of unusual non classical apoptosis inducers from large libraries in less time.

## Supporting Information

Movie S1 **Visualization of FRET loss over time.** MCF7 SCAT3 cells grown on chambered cover glass was treated with Camptothecin and imaged every 10 minutes after drug addition in controlled temperature and humidity conditions. Imaging was done using Nikon inverted fluorescent microscope under 20× NA 1.0 objective and Andor 885 camera. The frame rate is 30 frames per second.
(AVI)

Movie S2 **Simultaneous visualization of TMRM loss and caspase activation.** MCF7 SCAT3 cells stained with MMP indicator dye TMRM was treated with Actinomycin D and imaged every 10 minutes after drug addition. Imaging conditions are same as mentioned in Supplementary Movie S1. The frame rate is 30 frames per second.
(AVI)

Figure S1 **Visualization of FRET loss over time by widefield ratio imaging.** Images of Camptothecin treated MCF7 SCAT3 cells at 1 hr, 10 hr and 18 hr after drug addition taken from live cell imaging. Imaging conditions are same as mentioned in Supplementary Movie S1. Arrows indicate some representative cells that show FRET loss at the indicated time points.
(TIF)

Figure S2 **Simultaneous visualization of TMRM loss and caspase activation.** Confocal images of Actinomycin D treated MCF7 SCAT3 cells counterstained with TMRM for visualizing MMP loss at 4 hr, 16 hr and 20 hr after drug addition. Imaging conditions are same as mentioned in Supplementary Movie S1. Arrows indicate some representative cells that show FRET loss at the indicated time points.
(TIF)

Figure S3 **Validation of FRET sensor probe: Caspase inhibition reverses FRET loss pattern.** Panels A–D, E–H and I–L show Untreated, drug alone, Drug+inhibitor and Inhibitor alone treated cell populations of MCF7 SCAT3, SCAT8 and SCAT 9 cells respectively. Cells were subjected to drug treatment in the following manner: MCF7 SCAT3 and SCAT9 cells with Staurosporine (250 nM) and SCAT8 cells with Anisomycin (2 µg/ml) for 10 hours.
(TIF)

Figure S4 **Well positions of drugs** (Supplementary information to Figure 7) Well positions in 96 well format of the drugs used for kinetics study of caspase activation and chromatin condensation in HeLa SCAT3 cells. Detailed information on Drug concentrations used and the mechanism of action of drugs is given in Supplementary Table 2.
(TIF)

Table S1 **List of cell lines used in the study.**
(DOC)

Table S2 **Details of drugs used in the study.**
(DOC)
