# Supplementary figures and images for: High Throughput Ratio Imaging to Profile Caspase Activity: Potential Application in Multiparameter High Content Apoptosis Analysis and Drug Screening
Source: PLoS One. 2011 May 27;6(5):e20114. doi: 10.1371/journal.pone.0020114 (PMC3103529; doi:10.1371/journal.pone.0020114)

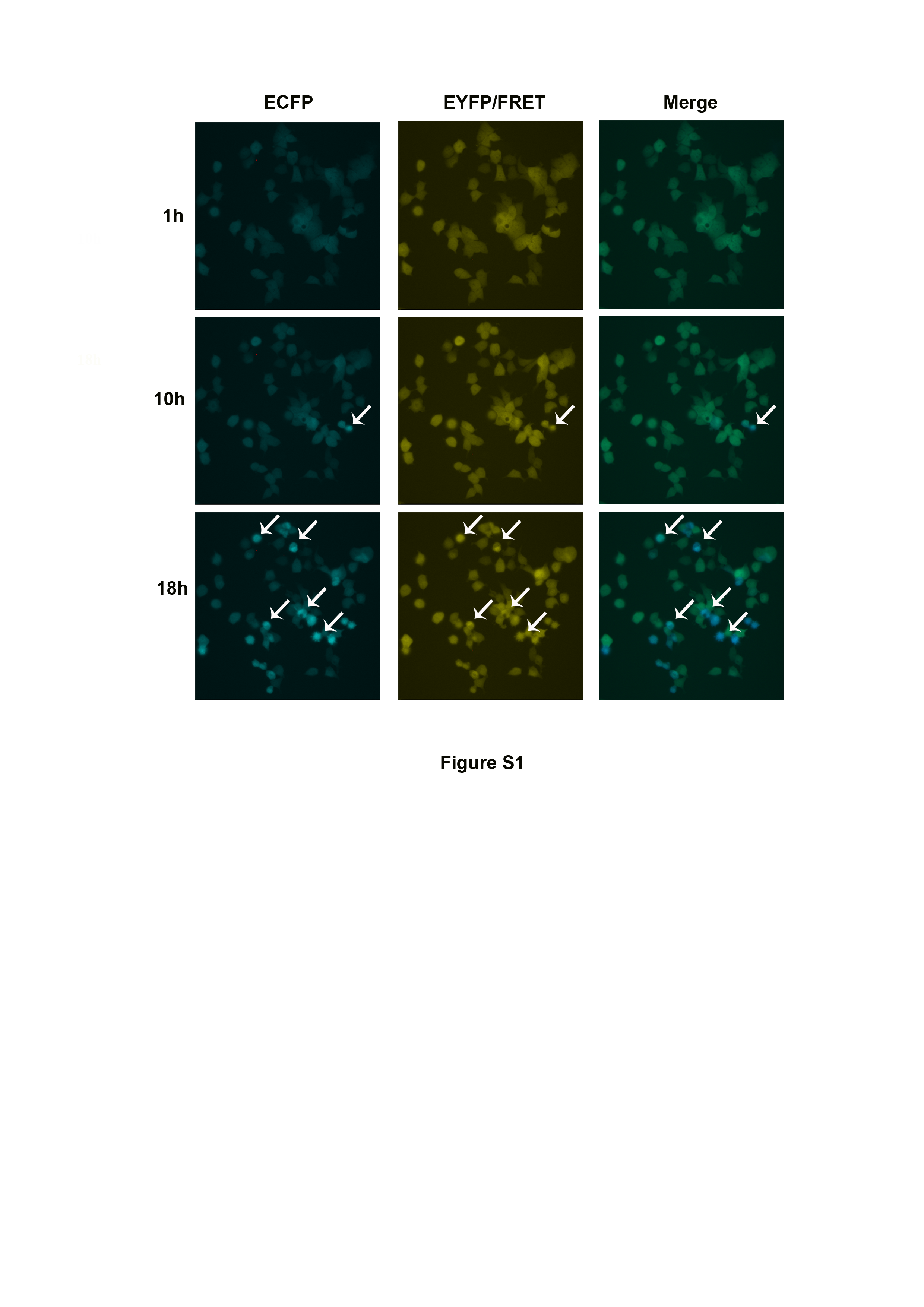

Supplement: Figure S1 — Visualization of FRET loss over time by widefield ratio imaging. Images of Camptothecin treated MCF7 SCAT3 cells at 1 hr, 10 hr and 18 hr after drug addition taken from live cell imaging. Imaging conditions are same as mentioned in Supplementary Movie S1. Arrows indicate some representative cells that show FRET loss at the indicated time points. (TIF) [file pone.0020114.s003.tif]

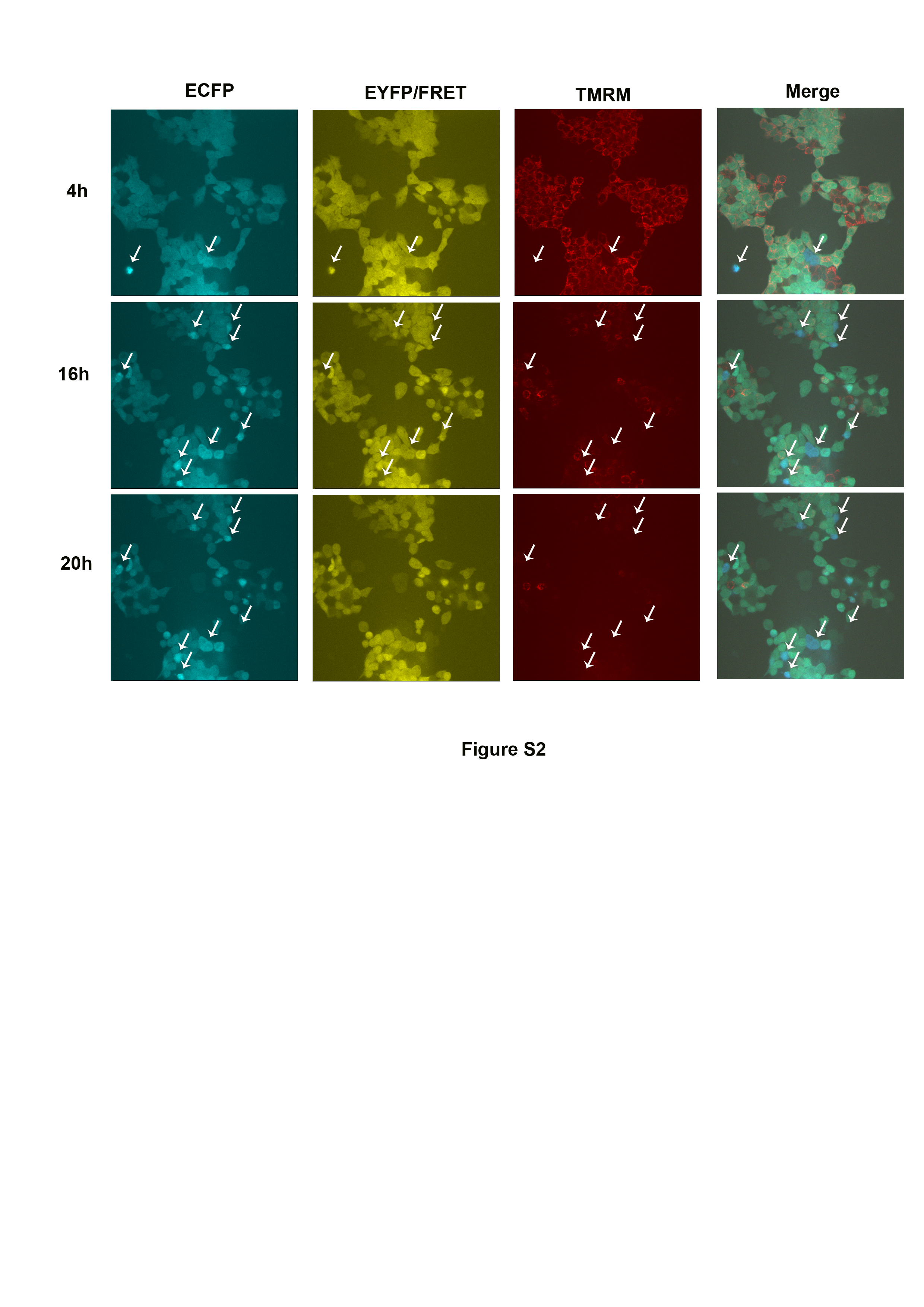

Supplement: Figure S2 — Simultaneous visualization of TMRM loss and caspase activation. Confocal images of Actinomycin D treated MCF7 SCAT3 cells counterstained with TMRM for visualizing MMP loss at 4 hr, 16 hr and 20 hr after drug addition. Imaging conditions are same as mentioned in Supplementary Movie S1. Arrows indicate some representative cells that show FRET loss at the indicated time points. (TIF) [file pone.0020114.s004.tif]

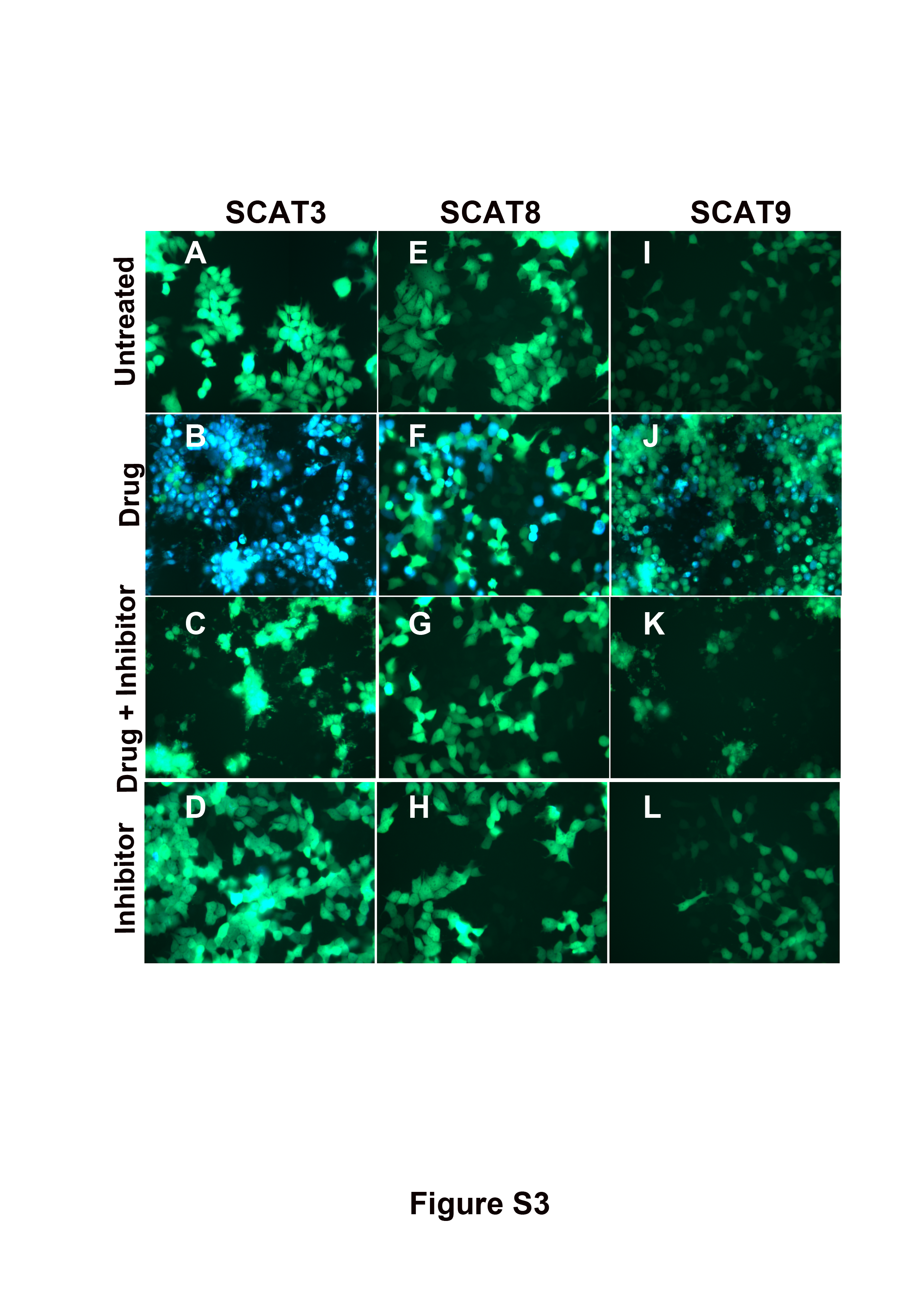

Supplement: Figure S3 — Validation of FRET sensor probe: Caspase inhibition reverses FRET loss pattern. Panels A–D, E–H and I–L show Untreated, drug alone, Drug+inhibitor and Inhibitor alone treated cell populations of MCF7 SCAT3, SCAT8 and SCAT 9 cells respectively. Cells were subjected to drug treatment in the following manner: MCF7 SCAT3 and SCAT9 cells with Staurosporine (250 nM) and SCAT8 cells with Anisomycin (2 µg/ml) for 10 hours. (TIF) [file pone.0020114.s005.tif]

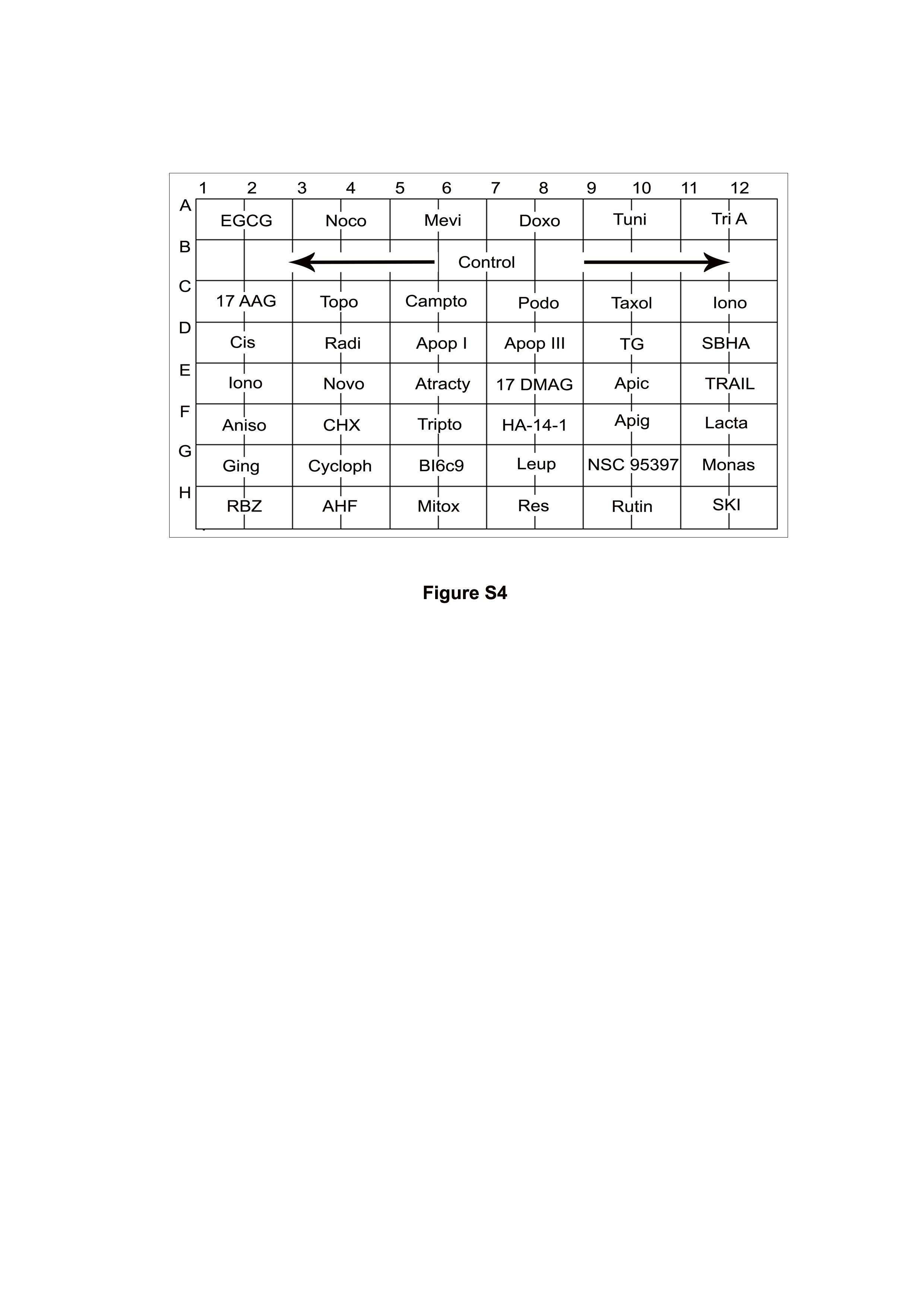

Supplement: Figure S4 — Well positions of drugs (Supplementary information to Figure 7) Well positions in 96 well format of the drugs used for kinetics study of caspase activation and chromatin condensation in HeLa SCAT3 cells. Detailed information on Drug concentrations used and the mechanism of action of drugs is given in Supplementary Table 2. (TIF) [file pone.0020114.s006.tif]
