# Supplementary material for: High Throughput Ratio Imaging to Profile Caspase Activity: Potential Application in Multiparameter High Content Apoptosis Analysis and Drug Screening
Source: PLoS One. 2011 May 27;6(5):e20114. doi: 10.1371/journal.pone.0020114 (PMC3103529; doi:10.1371/journal.pone.0020114)
Supplement: Table S1 — List of cell lines used in the study. (DOC) [file pone.0020114.s007.doc]

**Image based real time highcontent ratio imaging to identify caspase activating compounds: potential application in multiparameter apoptosis analysis and drug screening**

| **Sl no.** | **Compound** | **Abbreviation used** | **Possible Mechanism of action** | **Working concentration** |
| --- | --- | --- | --- | --- |
| **1** | Actinomycin D | Acti D | Blocks DNA dependent RNA synthesis | 10 µM |
| **2** | 4-aminohydroxyflavone | AHF | P56lck inhibitor | 50 µM |
| **3** | Anisomycin | Aniso | Inhibits peptidyl transferase activity, thereby inhibits protein synthesis | 2 µg/ml |
| **4** | Apigenin | Apig | G2/M arrest, apoptosis probably through p53, kinase inhibitor, inhibits Topo I catalyzed DNA religation | 100 µM |
| **5** | Apicidin | Apic | HDAC Inihbitor | 500 nM |
| **6** | Apoptosis Activator I | Apop I | selectively promotes apoptosome formation by antagonizing prothymosin-α (ProT), a negative regulator of mitochondria-initiated caspase activation | 200 µM |
| **7** | Apoptosis Activator II | Apop II | activates caspases in a cytochrome *c*-dependent manner and induces apoptosis by promoting Apaf-1 oligomerization | 20 µM |
| **8** | Apoptosis Activator III | Apop III | Embelin, Antagonizes XIAP | 50 µM |
| **9** | Atractyloside | Atracty | Inhibits ETC, toxic | 10 µM |
| **10** | BI6c9 | BI6c9 | Small molecule Bid inhibitor | 10µM |
| **11** | Caffeine | Caff | Lowers glutathione levels, CNS stimulant | 50 µM |
| **12** | Camptothecin | Campto | Irreversibly binds to DNA-Topo I complex, inhibiting DNA reassociation | 1 µM |
| **13** | CDK Inhibitor | CDKIn | CDK inhibition | 10 µM |
| **14** | Cisplatin | Cis | Cross-links DNA | 50 µg/ml |
| **15** | Colchicine | Colchi | Microtubule inhibitor | 50 µg/ml |
| **16** | Cycloheximide | CHX | Translation inhibitor | 250 µg/ml |
| **17** | Cyclophosphamide | Cycloph | DNA cross-linker | 100 µM |
| **18** | Doxorubicin | Doxo | Reverse transcriptase and RNA Pol inhibitor, DNA intercalator | 200 ng/ml |
| **19** | Epigallactocatechinegallate | EGCG | Inhibits VEGF-induced tyrosine phosphorylation | 125 µM |
| **20** | Gingerol | Ging | Anti-inflammatory, antitumor agent, inhibits COX-2 by blocking p38 MAPK and NF-κB | 500 µM |
| **21** | Gossypol | Gossy | Upregulates Fas/Fas L mediated apoptosis, Bax/Bak independent cytochrome c release, mediates free radical toxicity | 5 µM |
| **22** | HA-14-1 | HA-14-1 | Small molecule Bcl-2 antagonist | 100 µM |
| **23** | Hydroxyurea | HU | Inactivates entire replitase complex including Ribonucleotide reductase, thereby DNA synthesis | 2 µM |
| **24** | Indomethacin | Indo | COX-1 inhitor (general COX inhibitor) | 500 µM |
| **25** | Ionomycin | Iono | Ca^2+^ ionophore | 1 µM |
| **26** | Kaempferol | Kaemf | Nuclear DNA degradation, lipid peroxidation, Inhibits Topi – catalyzed DNA relegation | 100 µM |
| **27** | Lactacystin | Lacta | Proteasome inhibitor | 50 µM |
| **28** | Leupeptin | Leup | Serine and Cysteine Protease specifically, plasmin, trypsin, Papain and Cathepsin B | 50µ/ml |
| **29** | Licochalcone | Lico | Inhibits Topo I activity | 10 µM |
| **30** | Mevinolin | Mevi | HMG-CoA competitive inhibitor | 40 µM |
| **31** | MG132 | MG 132 | Proteasome inhibitor | 10 µM |
| **32** | Mimosine | Mimo | H_2_O_2_ formation, oxidative stress, mitochondrial inactivation | 200 µM |
| **33** | Mitoxantrone | Mitox | Intercalates DNA, DNA synthesis inhibitor | 1 µM |
| **34** | Monastrol | Monas | Mitosis inhibitor | 100 µM |
| **35** | Myricetin | Myr | α -glucosidase, glyoxalase I inhibitor | 250 µM |
| **36** | Nocodazole | Noco | Antimitotic agent, binds to β-tubulin | 100 nM |
| **37** | Novobiocin | Novo | Inhibitor of bacterial DNA gyrase and eukaryotic DNA topoisomerase | 500 µg/ml |
| **38** | NSC-95397 | NSC | Caspase 3 activation | 25 µM |
| **39** | PPIase Parvulin Inhibitor | ParvIn | Parvulin inhibitor | 20 µM |
| **40** | Pepstatin | Pep | Acid Protease (Pepsin, Renin, Cathepsin D) inhibitor | 200 µM |
| **41** | Pifithrin- α | Pif | Reversible inhibitor of p53-mediated apoptosis | 25 µM |
| **42** | Podophyllotoxin | Podo | Inhibits microtubule assembly | 10 µM |
| **43** | Radicicol | Radi | Antifungal macrolactone antibiotic, inhibits protein tyrosine kinase | 50 µM |
| **44** | 5,6-dicloro1,β-D ribofuranosyl benzimidazole | RBZ | inhibits RNA polymerase II-specific transcription | 100 µg/ml |
| **45** | Resveratrol | Res | Phenolic antioxidant, COX-1 inhibitor | 50 µM |
| **46** | Rutin | Rutin | Polyphenolic flavonoid, antioxidant, NO scavenger | 200 µM |
| **47** | SBHA | SBHA | HDAC inhibitor | 100 µg/ml |
| **48** | Sphingosine Kinase Inhibitor | SKI | Key enzyme in the generation of sphingosine 1-phosphate (S1P) which critically regulates  proliferation and migration | 2 µM |
| **49** | Staurosporine | Sta | Protein kinase inhibitor | 500 nM |
| **50** | Starvation^*^ | Starv | Known to trigger caspase activation and apoptosis | 0.5 % serum in culture medium |
| **51** | Sulindac | Sulin | MRP-1 and MRP-2 inducer | 100 µM |
| **52** | Thapsigargin | TG | Mobilizes intracellular Ca^2+^, ER stress inducer | 1 µM |
| **53** | Topotecan | Topo | Topo I inhibitor | 1 µM |
| **54** | TRAIL | TRAIL | Ligand to apoptosis inducing death receptors DR4 and DR5 | 200 ng/ml |
| **55** | Trichostatin A | Tri A | HDAC inhibitor | 1 µM |
| **56** | Triptolide | Tripto | Potentiated TNF α induction by suppression of c-IAP1 and c-IAP2 | 1 ng/ml |
| **57** | Tunicamycin | Tunica | Blocks protein N-glycosidic linkages | 50 µg/ml |
| **58** | Wortmannin | Wort | PI3K inhibitor | 50 nM |
| **59** | Vinblastin | Vinb | Microtubule inhibitor | 100 nM |
| **60** | 17 AAG | 17 AAG | Hsp90 inhibitor | 1 µM |
| **61** | 17 DMAG | 17 DMAG | Hsp90 inhibitor | 1 µM |
| **62** | 5-fluoruracil | 5 FU | Affects pyrimidine synthesis by inhibitmg thymidylate synthase | 100 µM |

**Table S1**
