# Supplementary material for: High Throughput Ratio Imaging to Profile Caspase Activity: Potential Application in Multiparameter High Content Apoptosis Analysis and Drug Screening
Source: PLoS One. 2011 May 27;6(5):e20114. doi: 10.1371/journal.pone.0020114 (PMC3103529; doi:10.1371/journal.pone.0020114)
Supplement: Table S2 — Details of drugs used in the study. (DOC) [file pone.0020114.s008.doc]

**Supplemental data Table S2**

**List of cell lines used in the study**

| **Cell line** | **Origin** | **Detects activity** |
| --- | --- | --- |
| **MCF7 SCAT3** | Human breast adenocarcinoma | DEVDase |
| **MCF7 SCAT8** | Human breast adenocarcinoma | IETDase |
| **MCF7 SCAT9** | Human breast adenocarcinoma | LEHDase |
| **HeLa SCAT3** | Human cervical carcinoma | DEVDase |
| **HeLa SCAT8** | Human cervical carcinoma | IETDase |
| **HeLa SCAT9** | Human cervical carcinoma | LEHDase |
| **OVCAR8 SCAT3** | Human ovarian adenocarcinoma | DEVDase |
| **U251 SCAT3** | Human glioma | DEVDase |

**Supplementary Table S1**
